# Detection and quantification of microRNA in cerebral microdialysate

**DOI:** 10.1186/s12967-015-0505-1

**Published:** 2015-05-07

**Authors:** Søren Bache, Rune Rasmussen, Maria Rossing, Niels Risør Hammer, Marianne Juhler, Lennart Friis-Hansen, Finn Cilius Nielsen, Kirsten Møller

**Affiliations:** Department of Neuroanaesthesiology, The Neuroscience Centre, Copenhagen University Hospital, Copenhagen, Denmark; Department of Neurosurgery, The Neuroscience Centre, Rigshospitalet, Copenhagen University Hospital, Copenhagen, Denmark; Centre for Genomic Medicine, The Diagnostic Centre, Rigshospitalet, Copenhagen University Hospital, Copenhagen, Denmark; Present address: Department of Clinical Biochemistry, Naestved Sygehus, Naestved, Denmark

## Abstract

**Background:**

Secondary brain injury accounts for a major part of the morbidity and mortality in patients with spontaneous aneurysmal subarachnoid hemorrhage (SAH), but the pathogenesis and pathophysiology remain controversial. MicroRNAs (miRNAs) are important posttranscriptional regulators of complementary mRNA targets and have been implicated in the pathophysiology of other types of acute brain injury. Cerebral microdialysis is a promising tool to investigate these mechanisms. We hypothesized that miRNAs would be present in human cerebral microdialysate.

**Methods:**

RNA was extracted and miRNA profiles were established using high throughput real-time quantification PCR on the following material: 1) Microdialysate sampled *in vitro* from A) a solution of total RNA extracted from human brain, B) cerebrospinal fluid (CSF) from a neurologically healthy patient, and C) a patient with SAH; and 2) cerebral microdialysate and CSF sampled *in vivo* from two patients with SAH. MiRNAs were categorized according to their relative recovery (RR) and a pathway analysis was performed for miRNAs exhibiting a high RR *in vivo*.

**Results:**

Seventy-one of the 160 miRNAs detected in CSF were also found in *in vivo* microdialysate from SAH patients. Furthermore specific miRNAs consistently exhibited either a high or low RR in both *in vitro* and *in vivo* microdialysate. Analysis of repeatability showed lower analytical variation in microdialysate than in CSF.

**Conclusions:**

MiRNAs are detectable in cerebral microdialysate; a large group of miRNAs consistently showed a high RR in cerebral microdialysate. Measurement of cerebral interstitial miRNA concentrations may aid in the investigation of secondary brain injury in neurocritical conditions.

**Electronic supplementary material:**

The online version of this article (doi:10.1186/s12967-015-0505-1) contains supplementary material, which is available to authorized users.

## Background

Secondary brain injury accounts for a major part of the morbidity and mortality in patients with spontaneous aneurysmal subarachnoid hemorrhage (SAH) [1]. However, the cellular mechanisms leading to this complication are incompletely understood. Cerebral microdialysis, in which a catheter lined by a semipermeable membrane is perfused in order to sample fluid containing substances from the cerebral interstitial space, is a promising tool to investigate these mechanisms. Even so, the substances measured in the clinical setting today are largely limited to products of metabolism, such as glucose, lactate and pyruvate [2,3].

MicroRNAs (miRNAs) are a group of 22 nucleotides long, non-coding RNA molecules involved in posttranscriptional regulation of complementary mRNA targets [4]. MiRNAs are well conserved in animals and are highly tissue specific. They act intracellularly, are transported outside the cells in exosomes and may exist in stable forms in body fluids [5]. Specific miRNAs are implicated experimentally in neuronal apoptosis following acute cerebral ischemia [6,7] intracerebral hemorrhage [8] and are associated clinically with the severity of traumatic brain injury [9]. Measuring the interstitial concentration of specific miRNAs may provide valuable information on tissue function. We hypothesized that miRNAs are present in human cerebral interstitial fluid, are consistently filtered through the membrane of a cerebral microdialysis catheter, and hence, can also be detected in human cerebral microdialysate with low analytical variation. Thus, we aimed to develop and validate a method to measure miRNA expression in cerebral microdialysate.

## Methods

The protocol for this study was approved by the Danish Regional Scientific Ethics Committee of the Capital Region # H-3-2013-009 and registered on clinicaltrials.gov # NCT01791257. According to Danish law, the samples described below were obtained following informed consent by either the patient or by their next of kin and their general practitioner.

### Samples

**Figure 1:**
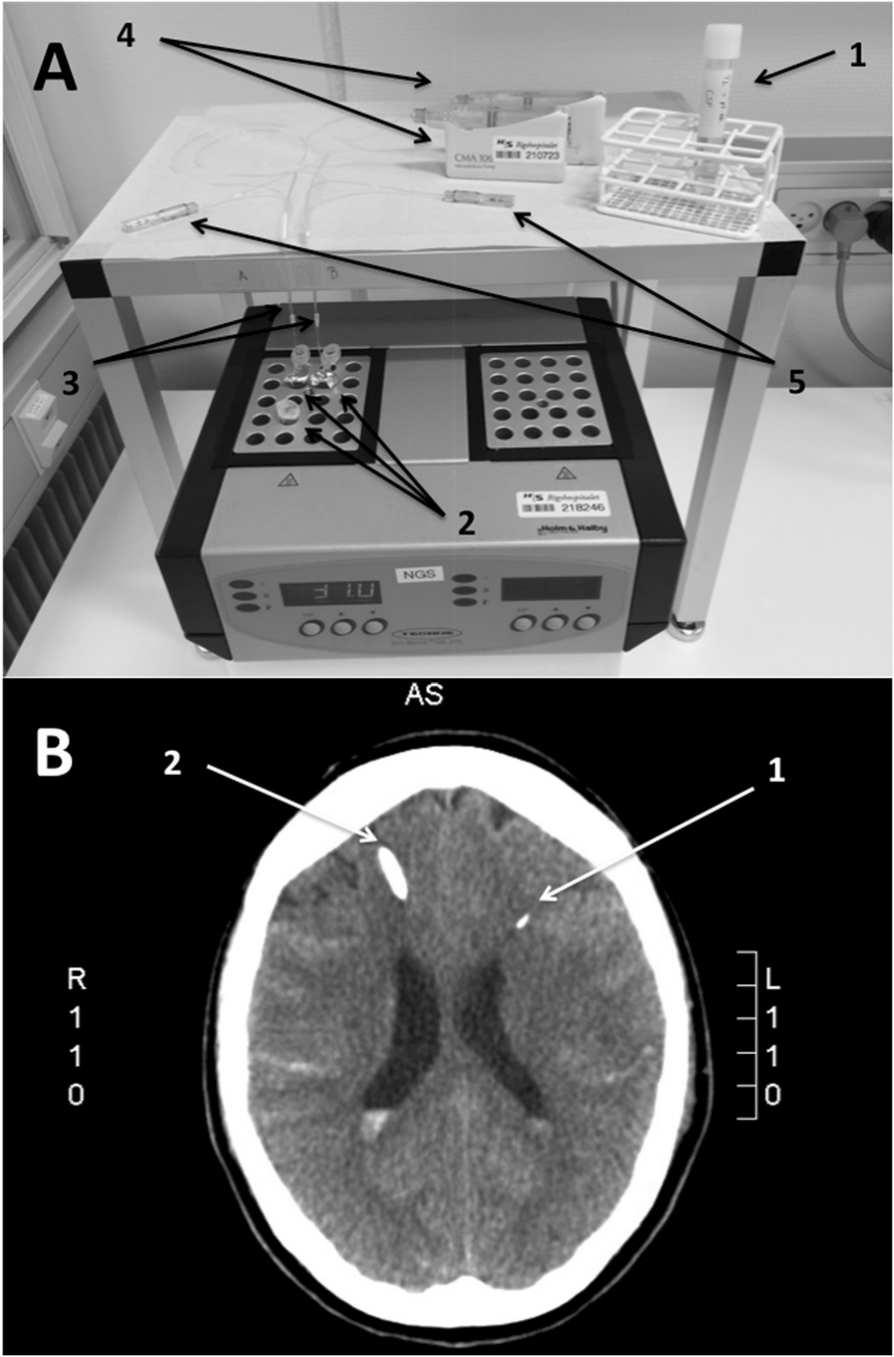
Materials and methods. A: Setup for *in vitro* microdialysis. 1, cerebrospinal fluid sample; 2, identical aliquots of the cerebrospinal fluid; 3, two microdialysis catheters with a membrane length of 10 mm and a 20 kDa cut-off; 4, perfusion pumps; 5, vials gathering samples of microdialysis fluid. B: Computed tomography scan from SAH patient 3. 1, a microdialysis catheter placed in the left frontal lobe; 2, part of the external drain passing through the brain.

In order to compare RNA in undialyzed samples to samples that had undergone microdialysis, human cerebral RNA, CSF and cerebral microdialysate was obtained and processed as follows:

1. Samples for *in vitro* studies:
  A. Total RNA extracted from human brain (Clontech Laboratories, Inc., California, USA) was stored at −80°C until use. Upon thawing, RNA was reconstituted in “CNS perfusion fluid” (MDialysis, Stockholm, Sweden) for a final volume of 3 ml and concentration of 21 μg/ml and divided into three aliquots. Samples underwent *in vitro* microdialysis as described below, using a catheter pore size of 20 or 100 kDa, or no microdialysis. The resulting samples were named “RNA MD20”, “RNA MD 100”, and “RNA REF”.
  B. CSF (2 ml) was aspirated using a 27G pencil point spinal needle prior to injection of local anesthetic in a neurologically healthy patient undergoing spinal anesthesia (hereafter referred to as a “healthy control patient”). The CSF was spun at 500 *g* for 10 minutes; the supernatant was stored at −80°C until use. Upon thawing and division into two aliquots, one sample underwent *in vitro* microdialysis as described below using a catheter pore size of 100 kDa, whereas the other sample was left undialyzed. The resulting samples were named “H MD100” and “H REF”.
  C. CSF (5 ml) was sampled from the external ventricular drainage system placed in a patient with SAH (patient 1) on day 6 after ictus. The CSF was spun at 500 *g* for 10 minutes; the supernatant was stored at -80°C until use. Upon thawing and division into five aliquots, four of these underwent *in vitro* microdialysis as described below, using four identical catheters with a pore size of 20 kDa, resulting in samples named “SAH1 MD20A through D”, whereas the remaining aliquot served as four undialyzed reference samples, named “SAH1 REFA through D”.
    Figure 1A depicts the *in vitro* study setup.
2. CSF and microdialysate samples for *in vivo* study:
  In two patients with SAH treated with external ventricular drainage and undergoing bedside microdialysis on clinical indications (patients 2 and 3), CSF from the drainage system as well as microdialysate from an intracerebral microdialysis catheter (Figure 1B) containing a 20 kDa membrane were collected on Day 3 and 9. This resulted in eight samples named “SAH2D3 MD20”, “SAH3D3 MD20”, “SAH2D9 MD20”, “SAH3D9 MD20”, “SAH2D3 REF”, “SAH3D3 REF”, “SAH2D9 REF”, and “SAH3D9 REF”.

Details regarding *in vitro* and *in vivo* samples are shown in Table 1. CSF samples were spun at 500 *g* for 10 minutes; the supernatant was stored at -80°C until use. Microdialysate was stored at -80°C until use.

**Table 1:** **Schematic overview of pre-analytical and analytical setup**

**Sample number**	**Sample name**	**Source**	**Micro-dialysis**	**Catheter pore size**	**Perfusion fluid for microdialysis**	**qPCR – number of miRNA**
1	RNA MD20	Human brain, total RNA	*In vitro*	20 kDa	CNS	377
2	RNA MD100	Human brain, total RNA	*In vitro*	100 kDa	HA 3.5%	377
3	RNA REF	Human brain, total RNA	None	NA	NA	377
4	H MD100	Healthy human, LP-CSF	*In vitro*	100 kDa	HA 3.5%	377
5	H REF	Healthy human, LP-CSF	None	NA	NA	377
6	SAH1 MD20A	SAH patient 1, EVD-CSF	*In vitro*	20 kDa	CNS	754
7	SAH1 MD20B	SAH patient 1, EVD-CSF	*In vitro*	20 kDa	CNS	754
8	SAH1 MD20C	SAH patient 1, EVD-CSF	*In vitro*	20 kDa	CNS	754
9	SAH1 MD20D	SAH patient 1, EVD-CSF	*In vitro*	20 kDa	CNS	754
10	SAH1 REFA	SAH patient 1, EVD-CSF	None	NA	NA	754
11	SAH1 REFB	SAH patient 1, EVD-CSF	None	NA	NA	754
12	SAH1 REFC	SAH patient 1, EVD-CSF	None	NA	NA	754
13	SAH1 REFD	SAH patient 1, EVD-CSF	None	NA	NA	754
14	SAH2D3 MD20	SAH patient 2, *in vivo* MD, day 3	*In vivo*	20 kDa	CNS	377
15	SAH3D3 MD20	SAH patient 3, *in vivo* MD, day 3	*In vivo*	20 kDa	CNS	377
16	SAH2D9 MD20	SAH patient 2, *in vivo* MD, day 9	*In vivo*	20 kDa	CNS	377
17	SAH3D9 MD20	SAH patient 3, *in vivo* MD, day 9	*In vivo*	20 kDa	CNS	377
18	SAH2D3 REF	SAH patient 2, EVD-CSF, day 3	None	NA	NA	377
19	SAH3D3 REF	SAH patient 3, EVD-CSF, day 3	None	NA	NA	377
20	SAH2D9 REF	SAH patient 2, EVD-CSF, day 9	None	NA	NA	377
21	SAH3D9 REF	SAH patient 3, EVD-CSF, day 9	None	NA	NA	377

qPCR, real-time polymerase chain reaction; miRNA, microRNA; MD, microdialysis; CNS, “Central nervous system perfusion fluid” (Mdialysis); HA, human albumin (CLS Behring); ref, reference; NA, not applied; LP-CSF, cerebrospinal fluid obtained by lumbar puncture; SAH, spontaneous aneurysmal subarachnoid hemorrhage; EVD-CSF = cerebrospinal fluid obtained from external ventricular drain.

### Microdialysis

*In vivo* and *in vitro* microdialysis was carried out using catheters (MDialysis, Stockholm, Sweden) with membrane lengths of 10 mm and cut-off values of either 20 kDa or 100 kDa as outlined above and in Table 1. Catheters were perfused at 0.3 μl min^−1^ with a CMA106 Microdialysis Pump (MDialysis) using “CNS perfusion fluid” (MDialysis) for 20 kDa membranes; for 100 kDa membranes, human albumin (3.5%; CLS Behring, Copenhagen, Denmark) was chosen because we observed fluid leakage across the 100 kDa membrane using “CNS perfusion fluid” as reported by others [10]. Vials for sampling were replaced every two hours, for a resulting volume of 36 μl per vial; for subsequent RNA isolation, no *in vivo* vials sampled within two hours of catheter insertion were used, the content of eight successive vials (288 μl) were pooled for each sample, and 200 μl was transferred for further processing as described below.

The temperature for all *in vitro* procedures was set at 37°C to resemble *in vivo* microdialysis. Undialyzed reference samples were kept at 37°C for two hours and stored at -80°C until use.

### RNA isolation

Total RNA was isolated from 200 μl of each sample according to the manufacturer’s protocol (Total RNA isolation kit, Appendix B, Norgen Biotek, Thorold, Canada), including the addition of beta-mercaptoethanol to the lysis solution.

### Real-time quantification

For each sample, a fixed volume of 6 μl eluted RNA sample was mixed on ice with 9 μl of a reverse transcription reaction containing 1.6 μl of 10X RT buffer, 1.8 μl of MgCl2, 0.2 μl of RNase-inhibitor (20 U/μl), 0.4 μl of dNTPs with dTTP (100 mM), 3.0 μl of Multiscribe Reverse Transcriptase (50U/μl), 1.6 μl of Megaplex RT primers (x10) and 0.4 μl of nuclease free water. Reverse transcription was performed using 40 cycles of 16°C, 2 min; 42°C, 1 min and 50°C, 1 sec followed by 85°C for 5 min and hold at 4°C. For each sample, a preamplification reaction comprising 62.5 μl of TaqMan PreAmp Master Mix (2X), 12.5 μl of Megaplex PreAmp Primers (10X), 37.5 μl of nuclease-free water and 12.5 μl of RT product were mixed on ice. After 10 min at 95°C, 2 min at 55°C and 2 min at 72°C the cDNA was subjected to 12 cycles of 15 sec at 95°C and 4 min at 60°C followed by 10 min at 99,9°C and hold at 4°C. The preamplified product was diluted with 375 μl of 0.1 TE buffer. For each sample, 450 μl of diluted preamplified product was mixed on ice with 450 μl of TaqMan Fast Advanced Master Mix and 800 μl loaded on a TaqMan Human MicroRNA Array Card. qRT-PCR was performed using the Viia 7 real-time PCR system (Applied Biosystems, Foster City, CA). Samples 6–13 were screened for 754 specific miRNAs using Human Pools A v.2.1 and B v.3.0. The remaining samples were screened for 377 specific miRNAs using Human Pools A v.2.1. Reagents and array cards were all purchased from Invitrogen, Carlsbad, CA.

### Normalization & statistical analysis

Cycle threshold was set to 0.1 and baseline between 3 and 15 for all targets. Cycle quantification (Cq) values correlate inversely with concentration. A Cq value >32 were considered below the detection limit and were discarded and the mean normalization strategy [11] was applied. To compare measurement variability in microdialysate and CSF, SDs were compared using the paired Student’s *t* test. P < 0.05 was considered significant.

MiRNAs were categorized according to their relative recovery (RR) in microdialysate as exhibiting a “high” or “low” RR according to the criteria described in Table 2; miRNAs, for which this characterization was not possible, were classified as having an “indeterminate” RR. Please refer to Table 2 for a detailed description of the criteria for categorization.

**Table 2:** **Criteria for categorization**

**Groups**	**Inclusion criteria for each specific miRNA**	**Based on samples**	**miRNA targets**
High RR	*in vitro* Cq_mean CSF_ – Cq_mean MD_ < ±2 and *in vitro* SD_CSF_ and SD_MD_ both < 1	6-13	63
		6-13	
Low RR	*in vitro* Cq_mean MD_ - Cq_mean CSF_ > 4 and *in vitro* SD_CSF_ < 1	6-13	53
		6-13	
Indetermined RR	Criteria not fulfilled	6-13	173
Not present	Not detected in any samples	6-13	465

Criteria for categorization of 754 screened miRNAs according to their relative recovery in microdialysate from a catheter with a membrane cut-off value of 20 kDa.

RR, relative recovery; Cq_mean CSF_, mean cycle quantification of the specific miRNA in sample 10-13, Table 1; Cq_mean MD_, mean cycle quantification of the specific miRNA in sample 6-9, Table 1; SD_CSF_, standard deviation of cycle quantification of the specific miRNA in sample 10-13, Table 1; SD_MD_, standard deviation of cycle quantification of the specific miRNA in sample 6-9, Table 1.

### MiRNA target pathway analysis

MiRNAs exhibiting a high RR *in vivo* were uploaded to the Ingenuity Pathway Analysis software (Qiagen, Venlo, Holland) and a search of putative mRNA targets was performed which was limited to “experimentally observed”. Target mRNAs were subsequently employed in a gene set enrichment analysis in the C2 Reactome Molecular Signature Database v4.0 (www.broadinstitute.org). A similar analysis was performed with a gene set of mRNAs that have been reported by others to be differentially expressed between the brain tissue from the perihematomal region and the contralateral region from patients suffering from intracerebral hemorrhage [12].

## Results

### MiRNA profiles of CSF and microdialysate

In the sample of total RNA extracted from human brain tissue, 205 out of 377 tested miRNAs (60%) were detected in the undialyzed reference sample; of these 205 miRNAs, 171 (83%) were detected after *in vitro* microdialysis using a membrane cutoff of 20 kDa, and 84 (41%) were detected after microdialysis using a 100 kDa membrane.

In the healthy control patient, 114 of 377 tested miRNAs (34%) were detected in undialyzed CSF; of these 114 miRNAs, 71 (62%) were detected after *in vitro* microdialysis using a 100 kDa membrane.

In the three patients with SAH, samples from patient 1 were screened for 754 specific miRNAs, whereas samples from patients 2 and 3 were screened for the presence of 377 miRNAs. In patient 1, 203 miRNAs were detected in all four identical samples of CSF, of which 112 (55%) were detected in all four corresponding microdialysate samples obtained *in vitro*. In patients 2 and 3, 160 miRNAs were detected in all four samples of CSF, and 71 (44%) of these were detected in all four samples of microdialysate obtained *in vivo*. Conversely, all miRNAs identified in all four *in vivo* samples of microdialysate were also identified in *in vivo* CSF. All mean normalized Cq values of specific miRNAs can be found in Additional file 1.

### Selective passage of microdialysis membrane

**Figure 2:**
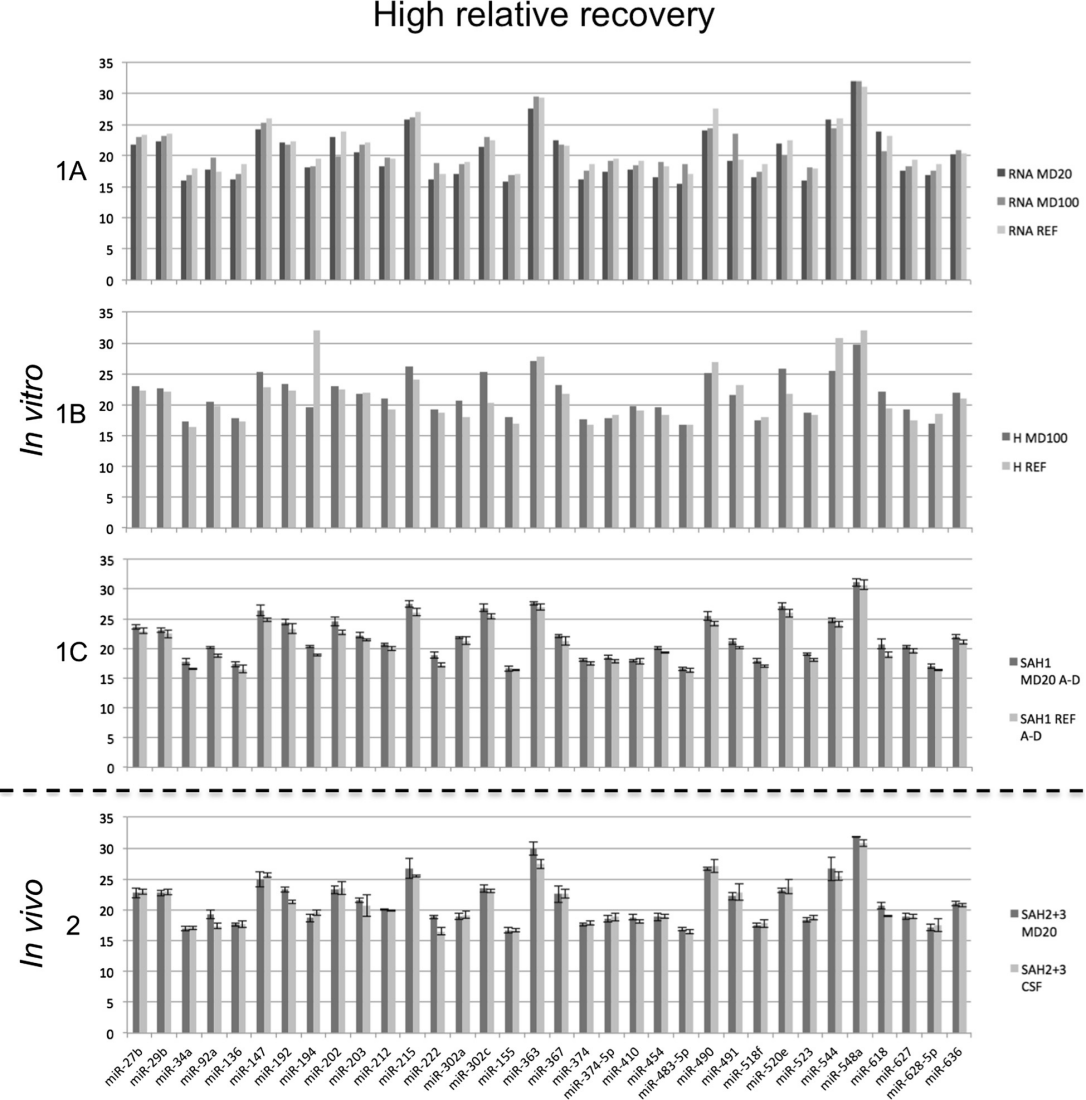
High relative recovery of miRNA. Mean normalized cycle quantification (Cq) for miRNAs showing a high relative recovery during microdialysis *in vitro* (1A, 1B, 1C) and *in vivo* (2). 1A: Cq values of miRNAs after *in vitro* microdialysis on total RNA from human brain. Dark grey column, 20 kDa membrane; grey column, 100 kDa membrane; light grey column, undialyzed reference sample. 1B: Cq values of miRNAs after *in vitro* microdialysis on CSF from a neurologically healthy patient. Dark grey column, 100 kDa membrane; light grey column, undialyzed reference sample. 1C: Mean Cq values of miRNAs after *in vitro* microdialysis on CSF obtained by lumbar puncture in a neurologically healthy patient. Dark grey column, 20 kDa membrane; light grey column, undialyzed reference sample. Error bars show SD (N of identical aliquots = 4). 2: Mean Cq values of miRNAs in *in vivo* microdialysate and CSF obtained from patients with subarachnoid hemorrhage. Dark grey column, *in vivo* microdialysate, 20 kDa membrane; light grey column, CSF, external ventricular drain. Error bars show SD (N of aliquots for each = 4). The four samples of each material was taken from two different patients with SAH on Day 3 and 9.

**Figure 3:**
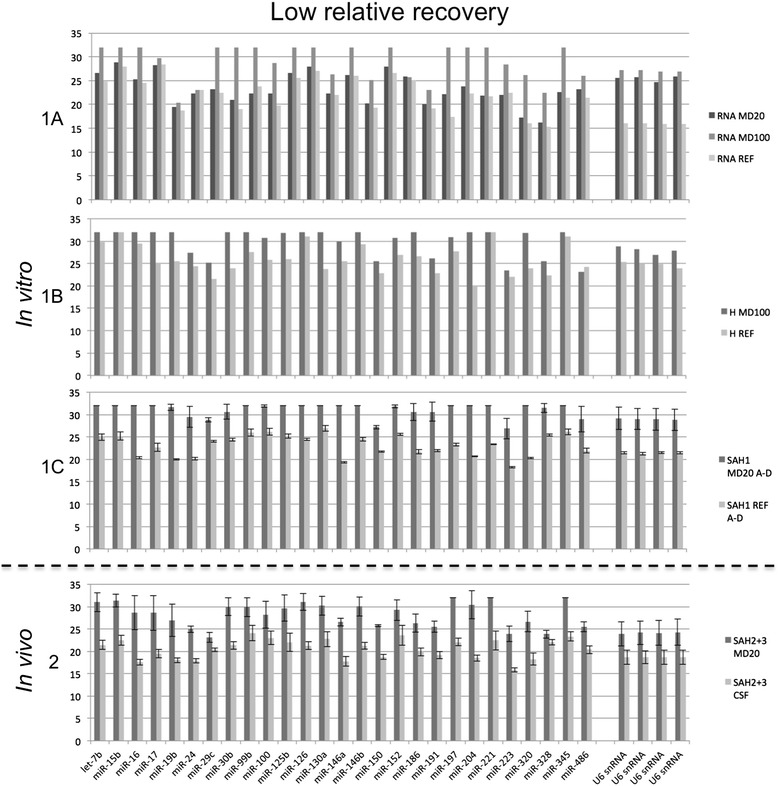
Low relative recovery of miRNA. Mean normalized cycle quantification (Cq) for miRNAs showing a low relative recovery during microdialysis *in vitro* (1A, 1B, 1C) and *in vivo* (2). 1A: Cq values of miRNAs after *in vitro* microdialysis on total RNA from human brain. Dark grey column, 20 kDa membrane; grey column, 100 kDa membrane; light grey column, undialyzed reference sample. 1B: Cq values of miRNAs after *in vitro* microdialysis on CSF from a neurologically healthy patient. Dark grey column, 100 kDa membrane; light grey column, undialyzed reference sample. 1C: Mean Cq values of miRNAs after *in vitro* microdialysis on CSF obtained by lumbar puncture in a neurologically healthy patient. Dark grey column, 20 kDa membrane; light grey column, undialyzed reference sample. Error bars show SD (N of identical aliquots = 4). 2: Mean Cq values of miRNAs in *in vivo* microdialysate and CSF obtained from patients with subarachnoid hemorrhage. Dark grey column, *in vivo* microdialysate, 20 kDa membrane; light grey column, CSF. Error bars show SD (N of aliquots for each = 4). The four samples of each material was taken from two different patients with SAH on Day 3 and 9.

The microdialysate relative recovery (RR) of different miRNAs varied considerably in the *in vitro* studies. However, the RR of each specific miRNA was consistent in the three *in vitro* studies. Of the 754 specific miRNAs screened, 63 were categorized as exhibiting a high RR, 53 as exhibiting a low RR, and 173 as exhibiting an indeterminate RR. The 465 remaining miRNAs were not identified in the reference material and therefore could not be evaluated. These characteristic abilities to pass through the membrane were reproduced in the *in vivo* studies. Figures 2 and 3 show Cq values of representative miRNAs from the group with a high RR and the one with a low RR and illustrates the reproducibility from *in vitro* to *in vivo* studies. The complete distribution of all 754 miRNAs in the four groups is shown in Additional file 2.

### Repeatability

In order to investigate analytical variation, we compared standard deviations of Cq values in microdialysate and CSF from patient 1 (SAH1 MD20A through D compared to samples SAH1 REFA through D, Table 1). Since miRNAs at low concentrations increase analytical variation in qRT-PCR markedly, including miRNAs with a low or indeterminate RR would lead to an overestimation of the analytical variation contributed by microdialysate. Therefore, analysis focused on the group of 63 miRNAs showing a high RR; the average SD of Cq values in microdialysate was lower than that of CSF (SD for microdialysate, 0.37 (95% CI, 0.33-0.42) vs. SD for CSF, 0.46 (95% CI, 0.41-0.51) (p = 0.011).

### MiRNA target pathway analysis

In order to obtain an overview of the possible biological significance of the 31 miRNAs exhibiting a high RR in microdialysate obtained *in vivo*, we performed a pathway analysis of putative target mRNAs. Transcripts were matched to the C2 Reactome in the Molecular Signature Database v4.0, and the 50 most significantly related reactomes (FDR q-value ≤ 1.02 × 10^−5^) are listed in Additional file 3. Comparing these results to the 50 most significantly related reactomes (FDR q-value ≤ 4.64 × 10^−3^) from a similar pathway analysis, based on a gene set of mRNAs differentially expressed between the brain tissue from the perihematomal region and the contralateral region from patients suffering from intracerebral hemorrhage, revealed an overlap of 13 reactomes (Additional file 3).

## Discussion

We have shown that miRNAs, important regulators of cell function, can be detected in human cerebral microdialysate sampled *in vivo*. Our findings have three main implications. First, this method offers a new approach to test hypotheses related to the presence, regulation and roles of miRNAs in the brain of neurocritically ill patients. Secondly, *in vivo* microdialysis for the study of miRNA is not limited to brain tissue, but could likely be applied to many tissues. Finally, the membrane of a microdialysis catheter resembles a semi-permeable membrane, and the results provide information on the much-debated topic of the size and characteristics of the proposed forms of miRNA which may prevent their degradation by RNases [13].

Besides showing the presence of miRNAs in cerebral microdialysis fluid sampled *in vivo*, the *in vitro* part of this study demonstrated that 63 out of 754 screened miRNAs show a high RR when using a 20 kDa microdialysis membrane. Of these 63 miRNAs, 34 were screened in the *in vivo* samples, and for 26 of these, a high RR was confirmed using the same criteria as for the *in vitro* study (Figure 2 - bottom). Of the remaining eight miRNAs, seven miRNAs came very close to meeting the criteria for a high RR; only one miRNA showed a markedly different expression pattern and variation. The reason for this outlying result is unknown, but could be due to both analytical imprecision or biological variation within samples. Nevertheless, the *in vivo* confirmation of the *in vitro* results shows consistency in the ability of each given miRNA to filter through the microdialysis membrane. Therefore, we recommend that future studies may include 62 of the 63 miRNAs meeting the criteria for a high RR, since their filtration fraction may be assumed to be stable as well.

The cut-offs for a given miRNA to be placed in the “high RR” group, an SD < 1 Cq and a microdialysate to CSF difference of < 2 Cq, was a compromise between increasing specificity by lowering both cut-offs and the challenge that each SD represents the qRT-PCR of one well from each of four array cards in order to allow for a wide miRNA-screening. This approach, however, means that the analytical variation cannot be reduced by running multiplicates (e.g.triplicates) of each miRNA. In our opinion, this may be a suitable approach when studying limited amounts of specific miRNAs of interest.

In order to compare samples obtained from different patients, at different time points, we estimated the analytical variation. For the 63 miRNAs showing a high RR, the average variability of expression values was significantly lower in microdialysate than in CSF. One explanation could be that microdialysis removes larger molecules, which interferes with downstream analysis or parts thereof (e.g. RNA extraction) in the undialyzed control samples. Nevertheless, the observation that microdialysis may not decrease, but in fact seems to increase, reproducibility of qRT-PCR, is important. Thus, microdialysis may provide a more accurate method than simple CSF sampling to monitor changes in cerebral miRNA expression over time and between patients and patient groups.

The above findings indicate that it may be possible to quantify the cerebral interstitial concentration of at least 62 miRNAs in a clinical setting with acceptable precision. Additionally, some miRNAs with an indeterminate or low RR appear to show acceptable measurement accuracy, though this would require additional studies. Finally, according to the experience in our laboratory, miRNAs may be quantified from as little as 25 μl of microdialysate, i.e. much less than used in this study, with good reproducibility (data not shown), indicating that continuous measurements with acceptable time resolution are possible in a clinical setting.

To our knowledge, this is the first time that *in vivo* microdialysis has been conducted in humans in order to sample miRNAs. For local changes in interstitial miRNA levels to be of importance, they need to reflect the intracellular levels. Accordingly, *in vitro* studies indicate that an energy-dependent equilibrium exists between the intracellular and extracellular compartments regarding miRNAs [14]. Furthermore, the miRNAs presented in Table 3 have previously been implicated in cerebral ischemia or inflammation. For instance, up-regulation of miR-29b in brain tissue following experimental transient middle cerebral artery occlusion, was found to inhibit Bcl2L2 gene expression resulting in increased neuronal cell death. In addition, inhibition of miR-29b increased Bcl2L2 gene expression and decreased neuronal cell death, indicating that miR-29b may be an important mediator of an adverse outcome in cerebral ischaemia [7]. In the present study, miR-29b showed a high RR in cerebral microdialysate. We suggest that changes in miR-29b may be monitored by microdialysis in humans in order to test the hypothesis raised in the study described above. Similarly, miR-34a and miR-155, which have been implicated experimentally in traumatic brain injury [15] and central nervous system inflammation following cerebral ischemia [16], both exhibited a high RR and therefore will be of great interest to monitor in similar patients. More generally, we compared results of pathway analyses of our data set of miRNAs exhibiting a high RR *in vivo* and a data set from patients with intracerebral hemorrhage. These analyses indicate that miRNAs exhibiting a high RR *in vivo*, may be implicated in the regulation of transcripts involved in among others hemostasis; axon guidance; signaling by nerve growth factor; adaptive immune system; platelet activation, signaling and aggregation; following intracerebral hemorrhage.

**Table 3:** **Selected references reporting roles of miRNAs with a high relative recovery during cerebral microdialysis**

**MiRNA**	**Author, year**	**Design/model**	**Tissue/material**	**Relation of miRNA to acute cerebral injury**
miR-29b	Shi et al, 2011 [7]	A: 90 min of MCA occlusion in rats (ischemia)	A: Brain tissue 24 hours after occlusion	Increased miR expression (A+B), repression of the anti-apoptotic protein Bcl2L2, and thereby neuronal cell death (B).
		B: Oxygen-glucose deprivation in cortical neuronal cell cultures from rats.	B: Cell suspension	Pre-treating neuronal cultures with miR-29b inhibitor decreased neuronal cell death (B)
miR-34a	Su et al, 2014 [16]	A: 15 min of MCA occlusion in mice (preconditioning)	A: Microglia extracted *ex vivo* after 3 days	MiR promotes CNS inflammation in microglia by suppressing transcription of the twist2 gene and thereby the anti-inflammatory gene cMaf.
		B: Microglia cell culture from mice	B: Cell suspension	Induced by expression of p53.
	Truettner et al, 2013 [15]	Stretch injury, cortical neuron cultures from rats	Cell suspension	MiR promotes apoptosis by inhibiting translation of the anti-apoptotic proteins Bcl2 and XIAP and increasing expression of the apoptotic cytokine Caspase 11.
miR-155	Su et al, 2014 [16]	A: 15 min of MCA occlusion in mice. (preconditioning)	A: Microglia extracted *ex vivo* after 3 days	Increased miR expression (A) MiR promotes CNS inflammation in microglia by suppressing the anti-inflammatory gene cMaf in microglia (A+B)
		B: Microglial cell culture from mice	B: Cell suspension	Normal cytokine induced expression of miR-155 is suppressed in p53-deficient microglia (B).
				Following treatment with INF-γ, the normal expression of IL-1α and IL-1β are suppressed in miR-155 knock out microglia cell cultures (B).
	Freilich et al, 2013 [19]	LPS stimulation, microglial cell cultures from mice pups	Cell suspension	Increased miR expression after LPS and thereby activation of several pro-inflammatory pathways.

MCA = middle cerebral artery, CNS = central nervous system, LPS = lipopolysaccharide.

The array cards used for miRNA screening were not designed to test specifically for miRNAs located in the brain and thus included a large number of miRNAs derived from other tissues. This is probably the reason for the 465 undetectable miRNAs in CSF and microdialysate. Regarding miRNAs detected solely in CSF and not in microdialysate, the molecular weight of a miRNA is around 6.5 kDa, but there is evidence that miRNAs acquire their extracellular stability from association in microvesicles / exosomes or by forming a ribonucleoprotein complex with Argonaute2, thus achieving protection against degradation by RNases [13,14,17]. Microvesicles have a diameter of 100–1000 nm, exosomes have a diameter of 30–100 nm, and Argonaute2 alone has a molecular weight of 96 kDa [17]. The microdialysis membranes used in the present study allows molecules with a size up to respectively 20 kDa and 100 kDa to pass through. Thus, microvesicles, exosomes and Argonaute2-bound miRNAs should not be able to pass through the membrane. By using differential centrifugation and size-exclusion chromatography, Arroyo et al. found miR-142-3p, −146b, −150, −193a-5p, −744 and −886-3p to be predominantly vesicle bound and miR-15b, 30c, 32, −126, and −191 to be at least in part vesicle-bound [13]. This is in agreement with data from our study, in which these miRNAs showed a low RR and none of the remaining predominantly vesicle-bound miRNAs were found in the group showing a high RR (Figures 2 and 3). Equally important, the above-mentioned vesicle-bound miRNA appeared to pass through the membrane when present as isolated RNA alone, i.e. without vesicle constituents or proteins (Figure 3-1A and Additional file 1). Furthermore, the observation that some miRNAs exhibit a high RR suggests that they exist at least partly in the free form in CSF, or that molecules smaller than Argonaute2 act as carriers in this medium.

The study has limitations regarding design and leaves room for further investigation. First of all, the *in vitro* study incubation at 37°C lasted on average 7 hours longer for samples used for microdialysis than for its identical reference samples. MiRNAs are considered stable in CSF at room temperature [18], but we cannot exclude that a fraction of miRNAs in the sample of total RNA from human brain, isolated from the constituents mediating RNase resistance, was degraded during this incubation period. Still, this would tend to reduce, rather than increase, the concentrations of miRNA in the *in vitro* microdialysate.

Secondly, as miRNA-profiling is still resource demanding, these studies are based on small sample sizes from few patients. We chose this approach in order to validate as many miRNAs as possible. Increasing reproducibility by increasing sample size would either increase costs significantly or we would have to measure only a few miRNAs. Likewise, we chose only to validate the *in vitro* data from screenings of 754 specific miRNAs with screenings of the 377 most common miRNAs (Human Pools A v.2.1) as the recovery results for these miRNAs could be extrapolated to the 377 miRNAs that were omitted (Human Pools B v.3.0).

Finally, we observed that miRNAs showed a higher RR through the membrane with a cut-off value of 20 kDa than with a cut-off value of 100 kDa. We speculate that the human albumin solution used as perfusion fluid for the microdialysis catheter, with a membrane cut-off value of 100 kDa, may have reduced the RR of miRNAs, either by repelling them or by somehow contributing to their degradation. At any rate, we found no indication of an increased RR of miRNA using a membrane with a cut-off value of 100 kDa which is why the remaining studies of miRNA in microdialysis was done using the 20 kDa membrane.

## Conclusions

We have identified a group of miRNAs that show a high relative recovery during microdialysis both *in vitro* and in a clinical setting. In the future, it may be relevant to study local changes in specific miRNAs in relation to brain tissue oxygen tension, cerebral metabolites and continuous electroencephalography as well as the clinical course of the patient.

## Additional files

Additional file 1:
**All normalized Cq values.** These four spreadsheets show normalized Cq values of all screened miRNAs from the *in vitro* study of microdialysis in the total RNA solution (1A), cerebrospinal fluid from a neurological healthy patient (1B), cerebrospinal fluid from a subarachnoid hemorrhage patient (1C) and normalized Cq values of all screened miRNAs from the *in vivo* study of microdialysis and cerebrospinal fluid from two subarachnoid hemorrhage patients sampled at day 3 and 9 (2A). Blanks represents a raw Cq value above 32. Additional file 2:
**All microRNA grouped according to relative recovery (RR).** This spreadsheet contains the 63 miRNA showing a high RR (column B), the 53 miRNA showing a low RR (column C), the 173 miRNA showing an indetermined RR (column D) and the 465 miRNA not detected (column E). Additional file 3:
**Pathway analysis of miRNAs exhibiting a high relative recovery (RR)** ***in vivo.*** The 50 most significantly related reactomes, including p-values and FDR q-values, from two gene set enrichment analyses in the C2 Reactome Molecular Signature Database v4.0 (www.broadinstitute.org) using the two target mRNA data sets derived as described below. *Left side of table –* A miRNA target filter analysis of the 31 miRNAs exhibiting a high relative recovery in microdialysate obtained *in vivo*. The analysis was conducted using the software Ingenuity Pathway Analysis (Qiagen, Venlo, Holland), and the search was limited to relationships that are “experimentally observed”. *Right side of table -* Gene set of mRNAs that have been reported to be differentially expressed in perihematomal tissue after spontaneous intracerebral haemoorhage [12]. RR, relative recovery; #, number of genes in gene set; GO, genes in overlap.
