# Supplementary material for: Detection and quantification of microRNA in cerebral microdialysate
Source: J Transl Med. 2015 May 7;13:149. doi: 10.1186/s12967-015-0505-1 (PMC4438475; doi:10.1186/s12967-015-0505-1)
Supplement: Additional file 3: — Pathway analysis of miRNAs exhibiting a high relative recovery (RR) in vivo. The 50 most significantly related reactomes, including p-values and FDR q-values, from two gene set enrichment analyses in the C2 Reactome Molecular Signature Database v4.0 (www.broadinstitute.org) using the two target mRNA data sets derived as described below. Left side of table – A miRNA target filter analysis of the 31 miRNAs exhibiting a high relative recovery in microdialysate obtained in vivo. The analysis was conducted using the software Ingenuity Pathway Analysis (Qiagen, Venlo, Holland), and the search was limited to relationships that are “experimentally observed”. Right side of table - Gene set of mRNAs that have been reported to be differentially expressed in perihematomal tissue after spontaneous intracerebral haemoorhage [12]. RR, relative recovery; #, number of genes in gene set; GO, genes in overlap. [file 12967_2015_505_MOESM3_ESM.pdf]

Putative mRNA targets of miRNAs exhibiting a high RR in microdialysate obtained in vivo						mRNA differentially expressed after intracerebral hemorrhage (Rosell et al)					
Rank	#	Genes involved in:	GO	p-value	FDR q-value	Rank	#	Genes involved in:	GO	p-value	FDR q-value
1	396	Developmental Biology	41	9.31E-33	6.27E-30	1	466	Hemostasis	38	3.7E-23	2.5E-20
2	122	Signaling by PDGF	16	3.77E-15	1.27E-12	2	933	Immune System	40	1.54E-14	5.18E-12
3	466	Hemostasis	26	1.44E-14	3.24E-12	3	91	Cell surface interactions at the vascular wall	14	4.04E-13	9.07E-11
4	251	Axon guidance	20	2.31E-14	3.28E-12	4	57	Chemokine receptors bind chemokines	10	2.44E-10	4.11E-8
5	137	Mitotic G1-G1/S phases	16	2.43E-14	3.28E-12	5	208	Platelet activation, signaling and aggregation	16	4,00E-10	5.4E-8
6	217	Signalling by NGF	18	2.35E-13	2.63E-11	6	396	Developmental Biology	21	7.5E-10	8.43E-8
7	38	G1 Phase	10	3.62E-13	3.48E-11	7	270	Cytokine Signaling in Immune system	17	2.46E-9	2.37E-7
8	148	Apoptosis	15	1.3E-12	1.09E-10	8	251	Axon guidance	16	6.09E-9	5.13E-7
9	933	Immune System	32	7.28E-12	5.45E-10	9	89	Response to elevated platelet cytosolic Ca2+	10	2.16E-8	1.62E-6
10	90	Signaling by ERBB4	12	8.81E-12	5.94E-10	10	188	Peptide ligand-binding receptors	13	6.26E-8	4.22E-6
11	38	PI3K events in ERBB4 signaling	9	1.59E-11	9.76E-10	11	159	Interferon Signaling	12	7.73E-8	4.74E-6
12	78	Signaling by SCF-KIT	11	3.5E-11	1.97E-9	12	79	Integrin cell surface interactions	9	9.78E-8	5.49E-6
13	421	Cell Cycle	21	4.21E-11	2.16E-9	13	63	Interferon gamma signaling	8	2.16E-7	1.12E-5
14	103	Signaling by NOTCH	12	4.48E-11	2.16E-9	14	120	Cell-Cell communication	10	3.76E-7	1.81E-5
15	18	Signaling by NODAL	7	5.77E-11	2.59E-9	15	305	Class A/1 (Rhodopsin-like receptors)	15	5.27E-7	2.37E-5
16	44	PI3K events in ERBB2 signaling	9	6.64E-11	2.8E-9	16	920	Signaling by GPCR	27	7.73E-7	3.26E-5
17	87	Extracellular matrix organization	11	1.18E-10	4.69E-9	17	408	GPCR ligand binding	17	9.4E-7	3.73E-5
18	208	Platelet activation, signaling and aggregation	15	1.72E-10	6.45E-9	18	539	Adaptive Immune System	19	2.6E-6	9.73E-5
19	325	Cell Cycle, Mitotic	18	1.98E-10	7.01E-9	19	195	G alpha (i) signalling events	11	4.73E-6	1.67E-4
20	127	Signaling by FGFR in disease	12	5.31E-10	1.79E-8	20	413	Transmembrane transport of small molecules	16	4.94E-6	1.67E-4
21	101	Signaling by ERBB2	11	6.07E-10	1.86E-8	21	70	Immunoregulatory interactions between a Lymphoid and	7	6.42E-6	2.06E-4
22	38	PI3K/AKT activation	8	6.08E-10	1.86E-8	22	133	Diabetes pathways	9	8.06E-6	2.47E-4
23	27	SMAD2/SMAD3:SMAD4 heterotrimer regulates	7	1.51E-9	4.44E-8	23	217	Signalling by NGF	11	1.3E-5	3.81E-4
24	63	Signaling by TGF-beta Receptor Complex	9	1.94E-9	5.45E-8	24	81	p75 NTR receptor-mediated signalling	7	1.69E-5	4.76E-4
25	17	Activation of BH3-only proteins	6	2.86E-9	7.71E-8	25	113	Signaling by Rho GTPases	8	1.85E-5	4.99E-4
26	30	Intrinsic Pathway for Apoptosis	7	3.4E-9	8.82E-8	26	805	GPCR downstream signaling	22	2.42E-5	6.27E-4
27	95	Downstream signal transduction	10	5.17E-9	1.29E-7	27	118	Toll Receptor Cascades	8	2.53E-5	6.32E-4
28	72	Transcriptional Regulation of White Adipocyte	9	6.55E-9	1.58E-7	28	279	Innate Immune System	12	2.72E-5	6.56E-4
29	100	Downstream signaling of activated FGFR	10	8.54E-9	1.99E-7	29	63	Signaling by TGF-beta Receptor Complex	6	3.94E-5	9.15E-4
30	56	PI-3K cascade	8	1.56E-8	3.41E-7	30	21	Antigen Presentation: Folding, assembly and peptide load	4	5.05E-5	1.13E-3
31	137	NGF signalling via TRKA from the plasma meml	11	1.57E-8	3.41E-7	31	132	Factors involved in megakaryocyte development and plat	8	5.65E-5	1.23E-3
32	109	Signaling by EGFR in Cancer	10	1.97E-8	3.96E-7	32	68	Semaphorin interactions	6	6.08E-5	1.28E-3
33	38	GAB1 signalosome	7	2,00E-08	3.96E-7	33	23	Signaling by BMP	4	7.36E-5	1.5E-3
34	38	Transcriptional activity of SMAD2/SMAD3:SMA	7	2,00E-08	3.96E-7	34	9	Endosomal/Vacuolar pathway	3	7.85E-5	1.56E-3
35	58	Collagen formation	8	2.07E-8	4,00E-07	35	74	G alpha (12/13) signalling events	6	9.79E-5	1.89E-3
36	112	Signaling by FGFR	10	2.57E-8	4.81E-7	36	11	Activation of Chaperone Genes by ATF6-alpha	3	1.52E-4	2.77E-3
37	25	G0 and Early G1	6	3.88E-8	7.07E-7	37	11	mTORC1-mediated signalling	3	1.52E-4	2.77E-3
38	64	NCAM signaling for neurite out-growth	8	4.6E-8	8.15E-7	38	241	SLC-mediated transmembrane transport	10	1.68E-4	2.99E-3
39	93	Activated TLR4 signalling	9	6.39E-8	1.1E-6	39	53	Circadian Clock	5	1.84E-4	3.12E-3
40	539	Adaptive Immune System	19	9.65E-8	1.63E-6	40	83	MyD88:Mal cascade initiated on plasma membrane	6	1.85E-4	3.12E-3
41	29	PIP3 activates AKT signaling	6	1.01E-7	1.67E-6	41	12	Platelet Adhesion to exposed collagen	3	2.01E-4	3.23E-3
42	74	TRIF mediated TLR3 signaling	8	1.46E-7	2.34E-6	42	12	Signal regulatory protein (SIRP) family interactions	3	2.01E-4	3.23E-3
43	31	GPVI-mediated activation cascade	6	1.55E-7	2.43E-6	43	30	Basigin interactions	4	2.15E-4	3.3E-3
44	352	Generic Transcription Pathway	15	2.03E-7	3.11E-6	44	30	Signaling by Robo receptor	4	2.15E-4	3.3E-3
45	107	Signaling by Interleukins	9	2.16E-7	3.23E-6	45	31	GPVI-mediated activation cascade	4	2.45E-4	3.68E-3
46	79	Integrin cell surface interactions	8	2.44E-7	3.58E-6	46	13	Activation of Chaperones by ATF6-alpha	3	2.59E-4	3.72E-3
47	81	p75 NTR receptor-mediated signalling	8	2.97E-7	4.26E-6	47	13	DCC mediated attractive signaling	3	2.59E-4	3.72E-3
48	118	Toll Receptor Cascades	9	4.98E-7	7,00E-06	48	32	Sema4D in semaphorin signaling	4	2.78E-4	3.91E-3
49	39	NCAM1 interactions	6	6.5E-7	8.95E-6	49	60	Cell death signalling via NRAGE, NRIF and NADE	5	3.31E-4	4.56E-3
50	40	Apoptotic cleavage of cellular proteins	6	7.6E-7	1.02E-5	50	93	Activated TLR4 signalling	6	3.44E-4	4.64E-3

Overlapping reactomes between the two gene set enrichment analyses are highlighted in red.
